# Pain Extent Is Not Associated with Sensory-Associated Symptoms, Cognitive or Psychological Variables in COVID-19 Survivors Suffering from Post-COVID Pain

**DOI:** 10.3390/jcm11154633

**Published:** 2022-08-08

**Authors:** César Fernández-de-las-Peñas, Stella Fuensalida-Novo, Ricardo Ortega-Santiago, Juan A. Valera-Calero, Corrado Cescon, Marco Derboni, Vincenzo Giuffrida, Marco Barbero

**Affiliations:** 1 Department of Physical Therapy, Occupational Therapy, Physical Medicine and Rehabilitation, Universidad Rey Juan Carlos (URJC), Alcorcón, 28922 Madrid, Spain; 2 VALTRADOFI Research Group, Department of Physiotherapy, Faculty of Health, Universidad Camilo José Cela, Villanueva de la Cañada, 28692 Madrid, Spain; 3 Rehabilitation Research Laboratory 2rLab, Department of Business Economics, Health and Social Care, University of Applied Sciences and Arts of Southern Switzerland, 6928 Manno, Switzerland; 4 Dalle Molle Institute for Artificial Intelligence (IDSIA USI-SUPSI), Department of Innovative Technologies, University of Applied Sciences and Arts of Southern Switzerland, 6900 Lugano, Switzerland

**Keywords:** COVID-19, pain extent, sensitization, post-COVID, pain

## Abstract

We aimed to investigate the relationship between pain extent, as a sign of sensitization, and sensory-related, cognitive and psychological variables in hospitalized COVID-19 survivors with post-COVID pain. One hundred and forty-six (67 males, 79 females) previously hospitalized COVID-19 survivors with post-COVID pain completed demographic (age, sex, height, weight), sensory-related (Central Sensitization Inventory, Self-Report Leeds Assessment of Neuropathic Symptoms), cognitive (Pain Catastrophizing Scale, Tampa Scale for Kinesiophobia) and psychological (Hospital Anxiety and Depression Scale, Pittsburgh Sleep Quality Index) variables. Pain extent and frequency maps were calculated from pain drawings using customized software. After conducting a correlation analysis to determine the relationships between variables, a stepwise linear regression model was performed to identify pain extent predictors, if available. Pain extent was significantly and weakly associated with pain intensity (r = −0.201, *p* = 0.014): the larger the pain extent, the lower the pain intensity. No other significant association was observed between pain extent and sensory-related, cognitive, or psychological variables in individuals with post-COVID pain. Females had higher pain intensity, more sensitization-associated symptoms, higher anxiety, lower sleep quality, and higher kinesiophobia levels than males. Sex differences correlation analyses revealed that pain extent was associated with pain intensity in males, but not in females. Pain extent was not associated with any of the measured variables and was also not related to the presence of sensitization-associated symptoms in our sample of COVID-19 survivors with long-term post-COVID pain.

## 1. Introduction

The world is facing a healthcare outbreak due to the Severe Acute Respiratory Syndrome Coronavirus-2 (SARS-CoV-2), the virus that causes the coronavirus disease, 2019 (COVID-19). Clinicians and researchers have used data from previous conditions, such as SARS, as a first step in better understanding SARS-CoV-2. Previous evidence shows that subjects who have survived SARS exhibit long-term symptoms, such as persistent fatigue, diffuse myalgia, weakness, depression, and nonrestorative sleep [1]. In fact, SARS survivors report a long-term substantial reduction in health-related quality of life, similar to those individuals who had survived other critical illnesses [2].

Due to the worldwide COVID-19-associated outbreak, thousands of publications about SARS-CoV-2 symptoms, looking at both the acute and post-acute phase, have been published. Current data have confirmed that subjects surviving SARS-CoV-2 also develop a plethora of long-term symptoms, e.g., fatigue, dyspnea, skin rashes, brain fog, sleep disorders, anxiety, and depression, after the acute phase of the infection [3,4,5]. Among this heterogeneous symptomatology, pain is also a common, but probably underestimated, post-COVID symptom. The prevalence of post-COVID pain ranges from 4.6% to 23.6% during the first six months after the infection, when this symptom is generally investigated in population-based studies [6]. However, the prevalence reaches 60% when pain is specifically investigated as a post-COVID symptom [7,8]. It seems that post-COVID pain has musculoskeletal pain features [9]. Since musculoskeletal chronic pain is associated with sensitization [10], it has been hypothesized that post-COVID pain could be also associated with altered nociceptive pain processing. Preliminary evidence suggests the presence of sensitization mechanisms in people with post-COVID pain [11,12,13].

Pain drawing is a patient-reported outcome measure (PROM), where patients draw their symptoms on paper or digital body charts, which are then used to assess the topographical distribution of pain. The quantification of pain extent is proposed as a potential outcome for identifying patients with sensitization, since wider-spreading pain is a clinical sign of altered nociceptive pain pathways [14]. In the last decade, several studies have investigated the association between pain extent and sensitization-associated variables in multiple chronic pain conditions [15]. This review identified that the association of pain extent with sensitization in clinical outcomes was different between musculoskeletal (e.g., neck pain, osteoarthritis) and non-musculoskeletal (e.g., neuropathic pain, or headaches) pain conditions [15]. No study has previously identified an association between pain extent and sensory associated variables in post-COVID pain. Our aim was to identify the correlation between large pain extent and sensory-related, sensitization-associated symptoms, cognitive and psychological variables in COVID-19 survivors with post-COVID pain. We also conducted a secondary analysis from a sex perspective to identify differences between male and female COVID-19 survivors with post-COVID pain.

## 2. Methods

### 2.1. Participants

This study included individuals who recovered from acute SARS-CoV-2 infection at three urban hospitals in Spain during the first wave of the pandemic. Inclusion criteria included: 1, diagnosis of acute SARS-CoV-2 infection by real-time reverse transcription polymerase chain reaction (RT-PCR) assay of nasopharyngeal or oral swab sample, and the presence of consistent clinical and radiological findings at hospitalization; 2, reporting “de novo” pain starting after the acute SARS-CoV-2 infection and lasting for at least three months; and 3, absence of any potential underlying medical condition that could best explain the pain, e.g., arthritis. Exclusion criteria included: 1, pre-existing history of pain symptoms before the acute infection; and 2, any other pre-existing medical comorbidity explaining pain symptoms. The study was approved by the Institutional Ethics Committees (INDIVAL 2020.416; HUIL/092-20, HSO 25112020; HUFA 20/126; URJC0907202015920). Patients were informed of the study procedure and each provided their written informed consent, prior to their inclusion.

### 2.2. Pain Extent

A set of two body charts (ventral and dorsal body views) representing both the male and female human body, printed on an A4 sheet, were provided to all participants. They were instructed to complete a pain drawing by shading with a red pencil, their pain symptoms. We specifically explained and instructed patients to shade the distribution of their pain symptoms as accurately as possible, regardless of the intensity and the type, avoiding the use of circle outlines or cross-marks. The reliability of this procedure has been previously confirmed [16].

The paper body charts were scanned into a pdf format and then imported into an online platform (https://syp.spslab.ch, accessed on 1 April 2022) which used a custom algorithm to count the number of pixels in each digital pain drawing. Pain extent was expressed as a percentage of the total body charts area (ventral: 476,650 pixels, dorsal: 489,592 pixels). Pain frequency maps were obtained by superimposing all of the patient pain drawings, in order to illustrate the most frequently reported location of pain across the sample. A color scale was used to indicate the percentage of people that reported pain in a specific area.

### 2.3. Sensory-Related Symptoms

Participants described the intensity of their usual pain symptoms on an 11-point numerical pain rate scale (NPRS, 0 to 10 points). Furthermore, the presence of sensitization-associated symptoms was evaluated with the Central Sensitization Inventory (CSI, score 0–100) [17]. Cut-offs >40 points suggests the presence of sensitization-associated symptoms [18]. The CSI has shown good psychometric properties in patients with persistent pain [19].

The Self-Report Leeds Assessment of Neuropathic Symptoms (S-LANSS, score 0–24) was used for evaluating the presence of neuropathic pain symptoms [20]. The S-LANSS uses binary responses, where patients confirm whether they suffer from several symptoms. A cut-off of ≥12 points suggests the presence of symptoms of neuropathic origin [20]. The S-LANSS has shown proper sensitivity, and good internal consistency and validity [20].

### 2.4. Cognitive Behaviour Variables

Pain catastrophizing, i.e., an exaggerated negative mental state brought to bear during an actual or anticipated painful experience, was assessed with the Pain Catastrophizing Scale (PCS, score 0–52) [21]. It includes 13 items (rated 0: never to 4: always), evaluating rumination, magnification, and despair aspects in relation to the pain experience.

Kinesiophobia, i.e., fear of movement, was assessed with the 11-item Tampa Scale Kinesiophobia (TSK-11, score 11–44) [22]. The TSK-11 includes 11 questions in which the patients choose how much they agree or disagree with each item (complete disagreement: 1 to complete agreement: 4) in relation to the presence of fear [22].

### 2.5. Psychological Variables

The Hospital Anxiety and Depression Scale (HADS) was used to evaluate anxiety (HADS-A, score 0–21) and levels of depression (HADS-D, score 0–21) [23]. Sleep quality was assessed with the Pittsburgh Sleep Quality Index (PSQI, score 0–21 points) [24]. The PSQI consists of 19 self-rated questions (rated from 0 to 3) assessing different aspects of sleep (e.g., usual bedtime, wake-up time, number of hours slept and time needed to fall asleep) during the previous month [24].

### 2.6. Sample Size Determination

Sample size calculation was based on the detection of significant small correlations (r = 0.15) between the variables with an alpha level (α) of 0.05 and a desired power (β) of 90%. This generated a sample size of at least 108 participants.

### 2.7. Statistical Analysis

Descriptive analyses (means and standard deviation -SD-) were used to describe the sample. The Shapiro-Wilk test revealed that quantitative data exhibited a normal distribution. Correlations between all the variables and the dependent variable (pain extent) were assessed with Pearson correlation coefficients (r_s_). This analysis was also used to identify multicollinearity and shared variance between the variables (r_s_ > 0.8). If statistically significant associations were identified, a stepwise multiple linear regression model (i.e., a hierarchical regression analysis) was conducted to identify the variables that significantly contributed to the variance in the pain extent, except for the variables showing multicollinearity. The significance criterion of the F value, for entry into the regression equation, was set at *p* < 0.05. Changes in adjusted *R*^2^ were reported after each step of the regression model to determine the association of the additional variables. Additionally, a sex-perspective analysis was conducted to separately determine differences between males and females (independent Student *t*-tests) and their associations with all the variables in males and females.

## 3. Results

**Figure 1:**
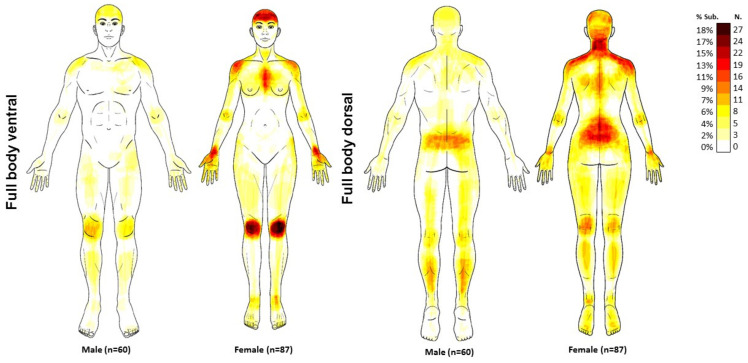
Pain frequency maps generated by superimposing the pain drawings of all COVID-19 survivors suffering from post-COVID pain (n = 147). The colour bar represents the frequency of coloured areas. Dark red indicates the most frequently reported area of pain.

Two hundred (n = 200) patients with long COVID were screened for participation. After verifying inclusion and exclusion criteria, 146 (73.5%) participants were included. Fifty-four participants were excluded because their main post-COVID symptom was fatigue or dyspnea, but not pain. Participants were assessed at a follow-up period of 18.8 ± 1.8 months after hospital discharge. Table 1 summarizes sensory-related, cognitive and psychological features of the sample, and by sex. Females exhibited a higher pain intensity, a higher CSI score, a higher HADS-A score, lower sleep quality and higher TSK-11 levels than males. Figure 1 illustrates pain frequency maps.

**Figure 2:**
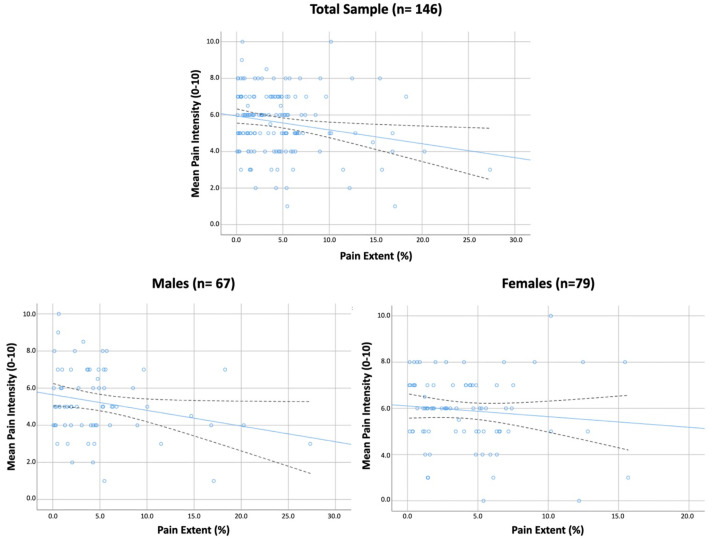
Scatter plots of correlations between pain extent and the intensity of pain symptoms in previously hospitalized COVID-19 survivors with post-COVID pain. Total sample (n = 146), males (n = 67); females (n = 79). Note that several points are overlapping. Blue lines represent adjusted lines and black lines represent 95% Confidence Intervals.

Overall, pain extent was only significantly weakly associated with pain intensity (r = −0.201, *p* = 0.014, Figure 2) in the total sample: the larger the pain extent, the lower the pain intensity. No other significant association was identified between pain extent and the remaining variables in the total sample. Table 2 shows Pearson’s correlation coefficients matrix in our sample of COVID-19 survivors with post-COVID pain by sex. The sex-perspective analysis revealed that the association between pain extent and pain intensity was significant, but still weak, in males (r = −0.242, *p* = 0.006), but not in females with post-COVID pain (Table 2, Figure 2).

## 4. Discussion

The current study found that pain extent was not associated with sensory-related, cognitive, or psychological variables in COVID-19 survivors with post-COVID pain. We only identified a weak (r < 0.3) association between large pain extent and lower intensity of pain symptoms in previously hospitalized COVID-19 survivors. This association was present in males, but not females, with post-COVID pain. Females reported higher levels of pain intensity, more sensitization-associated symptoms, higher anxiety levels, lower sleep quality and higher kinesiophobia levels when compared with males. 

The evaluation and quantification of pain drawings in the current study supports the idea that post-COVID pain tends to be widespread, with some areas being more frequently affected. In fact, the pain frequency maps revealed that the most-affected areas in our sample of COVID-19 survivors were the neck-shoulder, lower back, and knees, supporting that the idea that the widespread pain pattern is the sum of multiple localized pain areas, as was previously observed in people with fibromyalgia [25]. Supporting this similarity, it was found that almost 60% of COVID-19 survivors self-reported multiple pain sites [26], and that 30% of patients with post-COVID pain share common clinical features of fibromyalgia syndrome [27]. Accordingly, pain drawings would confirm that the pain pattern of post-COVID pain resembles the pattern of fibromyalgia syndrome. 

The presence of widespread pain symptoms as well as other associated symptoms would support the hypothesis that post-COVID pain has features of a “nociplastic” condition [28]. Several of the proinflammatory signaling molecules elevated in patients with COVID-19 due to the cytokine storm could impact skeletal muscle tissue. In such a scenario, a potential underlying mechanism explaining post-COVID pain would be that SARS-CoV-2 cell-to-cell inflammatory mechanisms (i.e., cytokine storms) lead to an excitability in the peripheral and central nervous systems throughout different pathways [29]. A long-lasting inflammatory state associated with the over-expression of cytokines would contribute to the development of post-COVID pain, due to the prolonged stimulation of nociceptive pathways [29]. This long-lasting stimulation of pain pathways would lead to the development of central sensitization, the basis for “nociplastic” pain [28]. In fact, it has become increasingly recognized that sensitization is likely an underlying mechanism of many chronic conditions, particularly musculoskeletal pain conditions [10]. The current results support that post-COVID pain could also be considered a nociplastic condition, although its classification as musculoskeletal or neuropathic remains controversial. It is probably true that a subgroup of patients with post-COVID pain would exhibit musculoskeletal pain features [9], whereas the other subgroup exhibits neuropathic features [30]. Nevertheless, discriminating between musculoskeletal, neuropathic, and nociplastic pain represents a current challenge, since one condition does not exclude the others [31].

In our study, pain extent was negatively but weakly associated with pain intensity, particularly in male patients with post-COVID pain, which was an unexpected finding. A higher intensity of symptoms (representing a peripheral process) has been associated with a larger pain extent (representing a central process) in other musculoskeletal pain conditions such as knee or hip osteoarthritis [15]. A potential association between pain intensity and pain extent would provide indirect evidence for the role of peripheral nociception in post-COVID pain; however, the presence of a negative relationship would suggest that pain intensity and pain extent represent two different mechanisms in this population. In fact, this association disappeared when adjusted by all variables in the regression model. These results are similar to those found in patients with migraine, tension-type headache, or carpal tunnel syndrome, but contrary to those previously observed in individuals with knee osteoarthritis or whiplash injury [15]. From an educational point of view, this “contradictory” association could be relevant for therapists when managing patients exhibiting post-COVID pain. Patients with chronic pain can expect that the more generalized the pain symptoms, the higher the intensity of pain; however, this situation could be different in patients with post-COVID pain. Accordingly, proper pain neuroscience education programs should integrate the findings observed in the current study. An alternative explanation for this negative association between pain intensity and pain extent could be an indirect effect of medication intake, a variable not evaluated in the current study. It is possible that COVID-19 survivors with higher pain intensity levels consume more pain medication. This hypothesis would explain the pain intensity mean scores in our sample of previously hospitalized COVID-19 survivors (<6/10 points), which could be considered low in comparison with other chronic pain populations. We do not know if subgrouping individuals with post-COVID pain according to the intensity of pain would reveal differences in sensitization-associated variables.

A larger pain extent is generally associated with sensitization; however, we did not find an association between pain extent and sensitization-associated symptoms. Previous studies in some musculoskeletal pain conditions, such as knee osteoarthritis, have found an association between pain extent and sensitization-associated symptoms [15]. It seems that the association of pain extent with other sensory variables is probably more evident in pain conditions of musculoskeletal origin, but less evident in neuropathic or nociplastic conditions. To date, post-COVID pain has not been classified as a musculoskeletal or neuropathic pain condition, explaining this lack of association. These results support the assumption that pain extent and patient-reported outcome measures, e.g., CSI or TSK-11, represent two dimensions of the sensory spectrum in post-COVID pain. Nevertheless, it is also possible that pain extent could be influenced by other factors, which are not included in this study. Finally, we also do not know if pain extent could be a predictor of future burden and related disability in individuals with post-COVID, as it has been recently observed in patients with chronic whiplash associated disorders at a follow-up period of two years [32]. Accordingly, any healthcare professional involved in the evaluation and management of people with post-COVID pain, e.g., medical doctors, physical therapists, or psychologists, should consider the integration of these aspects of the pain spectrum.

The last finding of this study was that females reported higher levels of pain intensity, more sensitization-associated symptoms, higher anxiety levels, lower sleep quality and higher kinesiophobia levels, compared to males. The fact that female sex is a risk factor for developing post-COVID symptomatology, including post-COVID pain, is not new in the literature [33]. Similarly, sex differences have been found in other chronic pain conditions, such as fibromyalgia, with a higher prevalence in females to males. Sex differences in pain perception [34] and cognitive/emotional processing [35] have been hypothesized to explain the current results. Our results support sex differences in some aspects of post-COVID, mostly in the psychological and cognitive aspects; however, due to the sample size, this comparison should be considered with caution at this stage. Sex differences in the psychological and cognitive aspects are supported by the fact that the association between pain extent and pain intensity was only significant in males and not females. 

Although this study used a pain-drawing software to eliminate estimation errors in extracting pain extent scores, other methodological issues should be recognized. First, we only collected data from previously hospitalized COVID-19 survivors. The analysis of pain extent from non-hospitalized COVID-19 survivors is still lacking. Second, this was a cross-sectional study; accordingly, we do not have data on the evolution of the pain extent in people with post-COVID pain. Third, although the procedure used has shown proper reliability in other pain conditions, reliability data in this population are still not available. Fourth, we did not collect psychophysical outcomes, e.g., quantitative sensory tests, to evaluate sensitization. We do not currently know if pain extent would be associated with these outcomes. Finally, we did not collect data on variations in pain intensity over a day, the previous week or previous month, which is an important sign of pain persistency, and, therefore, the development of chronic pain. 

## 5. Conclusions

This study has observed that pain extent was not associated with sensory-related, cognitive, or psychological variables in previously hospitalized COVID-19 survivors with post-COVID pain. A weak (r < 0.3) association was identified between larger pain extent and lower-intensity pain symptoms, but its relevance was minimal. Clinicians should consider this lack of association in the proper examination and understanding of post-COVID pain.

## Figures and Tables

**Table 1:** Demographic and clinical of the total sample (n = 146) and by sex (67 males; 79 females).

Variables	Total Sample	Males	Females	Sex Differences
Demographic Features				
Age (years) *	57.5 ± 11.8	60.1 ± 10.4	55.3 ± 12.4	4.8 (1.0; 8.6) *p* = 0.013
Height (m) *	1.67 ± 0.09	1.73 ± 0.09	1.61 ± 0.06	0.11 (0.08; 0.13) *p* < 0.001
Weight (kg) *	81.8 ± 17.1	86.5 ± 15.6	77.7 ± 17.3	8.8 (3.4; 14.2) *p* = 0.002
Body Mass Index (kg/m^2^)	29.2 ± 5.2	28.8 ± 4.5	29.6 ± 5.7	0.8 (−0.9; 2.5) *p* = 0.354
Sensory-Related Variables				
Pain intensity (NPRS, 0–10) *	5.6 ± 1.7	5.2 ± 1.9	5.9 ± 1.5	0.7 (0.1; 1.2) *p* = 0.017
Post-COVID Symptoms (months)	18.8 ± 1.8	18.7 ± 2.0	18.9 ± 1.7	0.2 (−0.4; 0.8) *p* = 0.484
S-LANSS (0–24)	7.4 ± 8.4	7.5 ± 10.5	7.2 ± 6.2	0.3 (−2.5; 3.1) *p* = 0.823
Central Sensitization Inventory (0–100) *	33.9 ± 17.25	25.9 ± 14.3	40.9 ± 16.5	15.0 (9.9; 20.1) *p* < 0.001
Psychological Variables				
HADS-A (0–21) *	5.3 ± 4.2	4.4 ± 4.0	6.0 ± 4.2	01.6 (0.2; 2.9) *p* = 0.022
HADS-D (0–21)	5.1 ± 4.3	4.4 ± 4.2	5.5 ± 4.3	1.1 (−0.3; 2.6) *p* = 0.111
Pittsburgh Sleeping Quality Index (0–21) *	8.1 ± 4.3	6.9 ± 4.4	9.0 ± 4.0	2.2 (0.8; 3.5) *p* = 0.002
Cognitive Behavior Variables				
Pain Catastrophizing Scale (0–52)	12.15 ± 11.95	10.3 ± 11.3	13.8 ± 12.4	3.5 (−0.4; 7.5) *p* = 0.080
Tampa Scale for Kinesiophobia (0–44) *	24.1 ± 8.55	22.6 ± 8.7	25.5 ± 8.3	2.9 (0.1; 5.7) *p* = 0.045

NPRS: Numerical Pain Rate Scale; HADS: Hospital Anxiety and Depression Scale (A: Anxiety; D: Depression); S-LANSS: self-reported version of the Leeds Assessment of Neuropathic Symptoms and Signs. * Statistically significant differences between males and females (Student *t*-test, *p* < 0.05).

**Table 2:** Pearson’s correlation coefficients matrix in COVID-19 survivors with post-COVID pain by gender.

Variables	1	2	3	4	5	6	7	8	9	10	11
Males (n = 67)											
1. Pain Extent											
2. Age	n.s.										
3. Body Mass Index	n.s.	n.s.									
4. Pain intensity	−0.242 *	n.s.	n.s.								
5. Post-COVID Symptoms	n.s.	n.s.	n.s.	n.s.							
6. S-LANSS	n.s.	n.s.	n.s.	n.s.	n.s.						
7. CSI	n.s.	n.s.	n.s.	n.s.	−0.255 *	n.s.					
8. HADS-A	n.s.	n.s.	n.s.	n.s.	−0.384 **	0.245 *	0.732 **				
9. HADS-D	n.s.	n.s.	n.s.	n.s.	n.s.	n.s.	0.528 **	0.851 **			
10. PSQI	n.s.	n.s.	n.s.	n.s.	−0.327 **	n.s.	n.s.	0.400 **	0.350 **		
11. PCS	n.s.	n.s.	n.s.	n.s.	−0.355 **	n.s.	0.501 **	0.578 **	0.571 **	0.298 *	
12. TSK-11	n.s.	n.s.	n.s.	n.s.	n.s.	n.s.	0.466 **	0.443 **	0.374 **	0.349 **	0.619 **
Females (n = 79)											
1. Pain Extent											
2. Age	n.s.										
3. Body Mass Index	n.s.	n.s.									
4. Pain intensity	n.s.	n.s.	n.s.								
5. Post-COVID Symptoms	n.s.	n.s.	n.s.	n.s.							
6. S-LANSS	n.s.	n.s.	n.s.	n.s.	−0.301 **						
7. CSI	n.s.	n.s.	n.s.	n.s.	n.s.	0.235 *					
8. HADS-A	n.s.	n.s.	n.s.	n.s.	n.s.	n.s.	0.375 **				
9. HADS-D	n.s.	0.244 *	n.s.	0.255 *	n.s.	n.s.	0.358 **	0.660 **			
10. PSQI	n.s.	0.273 *	n.s.	n.s.	n.s.	n.s.	0.416 **	n.s.	0.333 **		
11. PCS	n.s.	0.294 **	n.s.	n.s.	−0.361 **	n.s.	0.305 **	0.403 **	0.395 **	n.s.	
12. TSK-11	n.s.	n.s.	n.s.	n.s.	n.s.	0.285 *	0.388 **	0.237 *	n.s.	n.s.	0.529 **

Abbreviatures: CSI, Central Sensitization Inventory; PCS: Pain Catastrophizing Scale; PSQI, Pittsburgh Sleeping Quality Index; TSK-11, Tampa Scale for Kinesiophobia; * *p* < 0.05; ** *p* < 0.01; n.s. Non-significant.

## Data Availability

All data derived from this study are reported here.
